# Application of Hyperspectral Imaging and Deep Convolutional Neural Network for Freezing Damage Identification on Embryo and Endosperm Side of Single Corn Seed

**DOI:** 10.3390/foods14040659

**Published:** 2025-02-15

**Authors:** Jun Zhang, Limin Dai

**Affiliations:** 1 School of Mechanical and Electrical Engineering, Jiaxing Nanhu University, 572 Yuexiu South Road, Jiaxing 314001, China; 2 School of Agricultural Engineering, Jiangsu University, 301 Xuefu Road, Zhenjiang 212013, China; lmdai@ujs.edu.cn

**Keywords:** corn seed, freezing damage, endosperm and embryo, hyperspectral imaging, support vector machine, deep convolutional neural network, evaluation index

## Abstract

In this paper, the feasibility of identifying freezing damage on the endosperm side and embryo side of single corn seeds was studied by combining hyperspectral imaging technology and the deep convolutional neural network (DCNN) method. Firstly, hyperspectral image data of the endosperm and embryo side of three freezing-damage categories of corn seeds were collected, and the average spectra of the endosperm part and embryo part were obtained with the range of 450–979 nm. After the spectral data were pre-processed by non-pretreatment or standard normal variation (SNV) pretreatment, a support vector machine (SVM) and a DCNN model were established for freezing-damage identification. Finally, multiple evaluation indexes (including accuracy, sensitivity, specificity, and precision) were used to comprehensively evaluate the performance of the SVM and DCNN models in the whole waveband. The results showed that the DCNN model obtained better performance in accuracy, sensitivity, specificity, and accuracy. The values of each category, especially for the category-2 and category-3 testing sets of the SVM model, were lower than those of the DCNN model. The classification results of the embryo-side corn seeds were better than those of the endosperm side. The accuracy value of the testing set of the DCNN model on the embryo side was higher than 96.7%, while the accuracy value of the DCNN model on the endosperm side was lower than 93.8%. The specificity values of the SVM and DCNN models were both higher than 94%. In addition, the sensitivity and precision values of the category-2 testing set of the embryo-side DCNN model increased by at least 2.8% and 4.8%. The sensitivity value of the category-3 testing set of the DCNN model was improved by at least 8.2% and 4.4%. These results of the embryo side of the corn seed showed significant improvement in the training and testing set. This study proved that the DCNN model can accurately and quickly identify single freezing-damage corn seeds, which provided a theoretical basis for constructing an end-to-end recognition and classification model of frozen corn seeds.

## 1. Introduction

Corn (*Zea mays* L.), as one of the world’s three major food crops, plays a vital role in agricultural production and economic development [1]. Based on FAOSTAT data, global corn output reached 1.16 billion tons in 2022 (https://www.fao.org/faostat/zh/#data/QC). As the world’s second largest corn planting area and output country, China’s corn seed industry is mainly concentrated in the northwest and northeast regions. Due to the geographical location and seasonal climate characteristics of these areas, the seeds are vulnerable to cold waves and freezing damage. Freezing damage is a serious threat to the quality of maize seeds. There are studies indicating that freezing damage may lead to a significant decrease in seed germination rate, and the internal physiological structure of frozen seeds may be damaged [2,3]. In addition, freezing damage can also lead to decreased seed vitality, and even if it can germinate, its growth potential will be significantly limited [4,5]. Thus, seed freezing damage is one kind of agricultural disaster. Therefore, the detection of freezing-damaged corn seed is of great significance.

There are many limitations in the traditional methods of freezing damage detection. The appearance inspection can usually only detect the damage on the surface of the seed, and it is difficult to find the internal freezing damage. Moreover, the accuracy of the appearance inspection largely depends on the experience and subjective judgment of the inspector. Although the germination test can reflect the vitality of seeds to a certain extent, it takes a long time and cannot meet the needs of rapid detection. Zhang et al. (2022) applied NIRS to identify the frozen corn seeds; different preprocessing methods, feature extraction methods, and modeling methods were used. Although a 99.4% testing accuracy result was obtained, it is hard to determine the characteristics of individual corn seeds [3]. In addition, due to corn seed being composed of seed coat, endosperm, and embryo, the degree of frost damage may be different in different parts. For example, the embryo, as a key part of seed germination, is more sensitive to freezing damage, while the seed coat is relatively tolerant to freezing.

As a new detection technique with many unique advantages, hyperspectral imaging technology can obtain the spatial information and spectral information of the object at the same time, realize the multi-dimensional analysis of the sample, obtain the advantages of high resolution, high sensitivity, and non-destructive, and can quickly and accurately detect the chemical composition and physical characteristics of the object. It has a wide application in the non-destructive testing of agricultural products and foods in recent years [6,7,8,9], such as variety identification [10,11,12] and mycotoxin detection [13,14,15] of corn seeds, internal component testing of fruit [16,17], meat [18,19], etc. Referring to the relevant literature, the hyperspectral image data are analyzed and processed according to the following steps: Firstly, the region of interest and its spectral data are extracted by the image processing method. Then, pretreatment is usually conducted. Next, feature wavelength extraction and regression and classification analysis are generally adopted. According to the characteristics of hyperspectral imaging technology, it is applied to the detection of freezing damage of corn seeds, which may allow for the fine detection of different parts of seeds. By analyzing the spectral characteristics of different parts of seeds, the degree and location of freezing damage can be accurately judged. For example, when a certain part of the seed is subjected to freezing damage, its spectral characteristics will change accordingly, and through the analysis of these changes, the specific location and severity of freezing damage can be determined.

With the development of data processing methods, deep learning technology has shown remarkable effectiveness in the field of image recognition and data analysis [20,21,22]. In the field of agriculture, the application of deep learning technology has also been reflected. Convolutional neural network (CNN), as one of the most popular deep learning models, has a convolutional layer, a pooled layer, and a fully connected layer, which are responsible for feature extraction, feature dimensionality reduction, and classification, respectively. The combination of deep learning technology and hyperspectral imaging technology has certain applications in corn seed variety identification [23,24], mycotoxin detection [25,26] and so on. In view of the differences between the endosperm side and the embryo side of corn seeds, this study attempted to use hyperspectral imaging technology with a deep learning method to achieve rapid and accurate detection of freezing damage. Not only can the seed position be accurately located, but also through the training of a deep learning model, the automatic recognition and analysis of freezing damage features in hyperspectral images can be realized.

Through the literature review, there are only single-sided nondestructive testing studies on corn seed freezing damage detection. Nondestructive testing for different sides has not been studied, and this study will fill the gap of different side detection of corn seed freezing damage. The objective of this study was to explore the feasibility of identifying freezing damage on the endosperm side and embryo side of single corn seeds by combining the hyperspectral imaging technology and deep convolutional neural network. The conducted work was to (1) obtain the corn seed endosperm and embryo hyperspectral image; (2) develop the appropriate structure of the DCNN model; (3) establish the classification models based on SVM and DCNN with non-pretreatment or SNV pretreatment; and (4) compare and analyze the modeling performance with multiple evaluation indexes (accuracy, sensitivity, specificity, and precision).

## 2. Materials and Methods

### 2.1. Samples

The ‘Deyu 997′ (variety source: LK910 × LK122, weighing 36.5 g/100 grains; crude protein content is 9.54%, crude fat content is 4.05%, crude starch content is 71.7%, and lysine content is 0.30%) corn seeds with the initial water content of about 25% were collected from Changchun, Jilin Province, China. In order to obtain freezing damage samples, the corn seeds were placed under different freezing damage environments (unfrozen, −5 °C, −10 °C, −15 °C, −20 °C for 10 h) according to our previous study [27,28]. After the water content of the seeds was reduced to 13%, the endosperm-side and embryo-side hyperspectral image data were collected. A total number of 1920 corn seeds were divided into 3 categories (Unfrozen; slightly freeze-damaged samples frozen at −5 °C and −10 °C for 10 h; severely freeze-damaged samples frozen at −15 °C and −20 °C for 10 h). The number of seeds in each category was 800, 640, and 480, respectively. The data set of each category was divided into a training set and a testing set with the ratio of 2:1 by the KS algorithm [29]. Finally, the sample numbers of the training set were 533, 427, and 320, and the sample numbers of the testing set were 267, 213, and 160.

### 2.2. Hyperspectral Image Acquisition and Correction

The hyperspectral data were collected using a VIS/NIR hyperspectral imaging system (Dualix Spectral Imaging, Inc., Wuxi, China) in line-scan modes. The system consists of a CCD camera (C8484-05G, Hamamatsu Photonics, Hamamatsu City, Japan), an Impressor V10E-QE, Spectral Imaging Ltd., Oulu, Finland), a lens (V23-f/2.4030603, Specim Ltd., Oulu, Finland), a line light source (P/N9130, Illumination Technologies, Inc., New York, NY, USA), a light source controller (2900ER, Illumination Technologies, Inc., USA), a sample station (GZ02DS20, Guangzheng Instruments Co., Ltd., Beijing, China), a mobile platform and its controller (PSA200-11-X, Zolix Instruments Co., Ltd., Beijing, China), hyperspectral data acquisition software (SpectralCube V2.75b), and mobile platform mobile control software.

**Figure 1:**
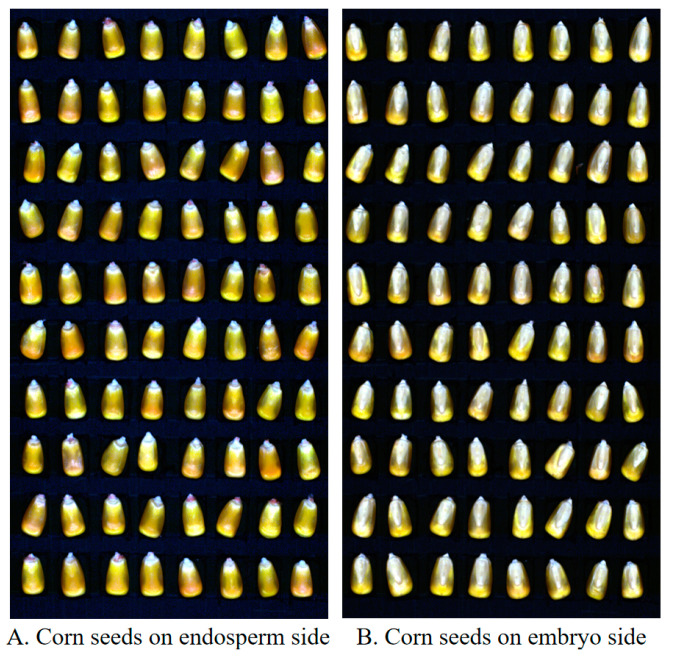
The obtained images of corn seeds (**A**): endosperm side; (**B**): embryo side.

Spectracube 2.75b (Spectral Imaging Ltd., Oulu, Finland) was used for image acquisition and correction. During the experiment, the corn seed endosperm and embryo were placed upside on the sample stage, respectively, with an object distance of 28.5 cm, and the moving speed of the mobile station was 2.6 mm/s. A 0.5 mm extension tube was added to obtain a clearer hyperspectral image of corn seeds. The hyperspectral data *I_o_* of different frozen corn seeds were collected in the range of 400–1000 nm, with a total of 477 wavelengths. Finally, a total of 48 hyperspectral images (24 for the endosperm side and 24 for the embryo side) were acquired. In each hyperspectral image, 80 corn kernels were used for data acquisition. Thus, 1920 corn seeds for the endosperm side and 1920 for the embryo side. Figure 1 shows the obtained images of corn seeds on the endosperm side (Figure 1A) and the embryo side (Figure 1B).

The hyperspectral image data were corrected according to Equation (1):

(1) $$I=\frac{I_{o}-I_{B}}{I_{W}-I_{B}}$$

where *I_B_* is the dark-field hyperspectral image data and *I_W_* is the Teflon white board hyperspectral image data.

### 2.3. Data Processing Methods

#### 2.3.1. Imaging Segmentation and Spectra Pretreatment

An ENVI 4.6.1 (ITT Visual Information Solutions, Boulder, CO, USA) was used for analyzing and processing hyperspectral image data. Image enhancement and the Otsu threshold segmentation on the gray image at 700 nm and 500 nm were conducted [15,27], and the endosperm and embryo hyperspectral images were obtained, respectively, according to our previous study. That is, the endosperm region of interest (ROIs) was separated from the background, and the embryo ROIs were separated from the endosperm part. Then, the endosperm average spectral data of the endosperm ROIs and the embryo spectral data of the embryo ROIs were conducted for subsequent spectral modeling. To eliminate the low response and high noise of the environment, the average spectrum in the 36–455th waveband range (from 450 to 979 nm) of each seed was finally selected for analysis. In this study, standard normal variation (SNV) was applied to eliminate interference by solid particle size, surface scattering effects, and optical path variations on spectral data [30].

#### 2.3.2. Classification Models and Evaluation Indexes

In this paper, the performance of the SVM and DCNN models was established for the classification of freeze-damaged corn seeds.

##### SVM

Support vector machine (SVM) is a supervised machine learning approach, widely used in spectral data classification [31]. The core goal is to find an optimal hyperplane in the feature space that separates different classes of data points. It tries to improve the generalization ability of the learning machine; the own optimization goal of SVM is to minimize structural risk rather than empirical risk. The original data are mapped into a high-dimensional space with an appropriate kernel function so as to effectively process nonlinear data so that the originally nonlinear data in a low-dimensional space becomes linear and divisible in a high-dimensional space, and then find the appropriate classification hyperplane. A kernel function is needed during the modeling process of the SVM model. As a commonly used kernel function, radial basis function (RBF) was applied in this study because it can make complex nonlinear spectral data become linearly separable or approximately linearly separable in high-dimensional space. Two main parameters, *c* (the penalty coefficient) and *g* (the radial width of the kernel function), were optimized by a grid search algorithm coupled with ten-fold cross-validation during the model development and updating stage.

##### DCNN

Convolutional neural network (CNN) is one of the core algorithms in the field of deep learning, and its architecture is mainly composed of a convolutional layer, a pooling layer, and a fully connected layer [28]. The convolution layer is responsible for feature extraction, and the convolution operation is carried out by applying filters to the input data. In the pooling layer, feature dimension reduction is performed to reduce the data dimension through downsampling while retaining key feature information. Common pooling methods include maximum pooling and average pooling. After the combination of multiple convolution layers and pooling layers, one or more fully connected layers are usually connected. The fully connected layer is to integrate the local features extracted by the convolutional layer to form the final output.

**Figure 2:**
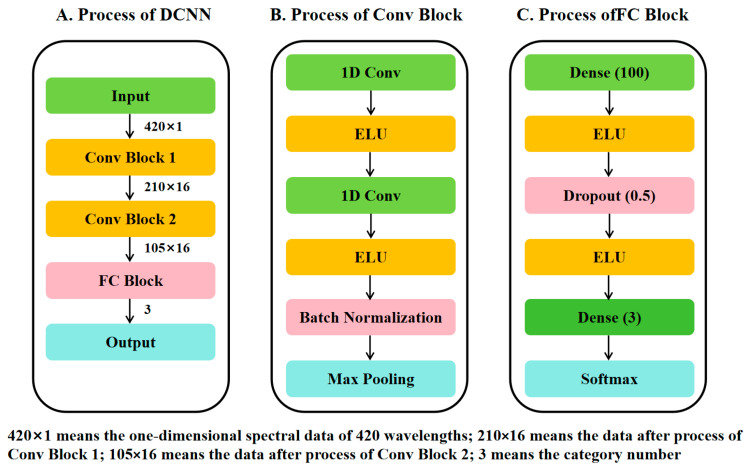
DCNN structure. (**A**) The main process of DCNN; (**B**) The specific process of Conv Block; (**C**). The specific process of FC Block.

The deep convolutional neural network structure adopted in this paper was shown in Figure 2, which was composed of two convolutional blocks (Conv Blocks) and one fully connected block (FC Block). The input data were the one-dimensional spectral data of 420 wavelengths (420 × 1); each convolutional block consisted of two convolutional layers and a maximum pooling layer. Each convolutional layer was configured with 16 filters, and the kernel size, step size, and fill of the filters were set to 3, 1, and 1. The maximum pooling layer with a size of 2 was adopted to halve the number of features. After the process of Conv Block 1 and Conv Block 2, the data were transferred to (210 × 16) and (105 × 16). While the fully connected block, as the end of the network, contained two Dense layers and one Dropout layer (dropout ratio was 0.5). The first Dense layer (the size was 100) was used to integrate the data of the two convolutional blocks, while the second Dense layer (the size was 3, which is the same as the category number) acted as the output layer, activated the output through the softmax function, and mapped the values into the range of 0–1. The exponential linear unit and softmax function were selected as activation functions, and batch normalization was applied to the convolutional block. The whole DCNN structure was trained for 200 epochs.

##### Performance Evaluation Index

In order to evaluate the classification performance of the SVM and DCNN models, the results were represented by a confusion matrix. In addition, accuracy, sensitivity, specificity, and precision were calculated [32,33]. For each index, the higher the value, the better the performance of the corresponding model.

(1) Accuracy refers to the ratio of the number of correctly classified samples to the total number of all samples and is calculated as follows:
  (2) $$Accuracy=\frac{\mathrm{TP}+\mathrm{TN}}{TP+TN+FP+FN}\times 100\%$$
(2) Sensitivity can evaluate the correct classification ability of the model for normal samples. It can be calculated as follows:
  (3) $$Sensitivity=\frac{\mathrm{TP}\;}{TP+FN}\times 100\%$$
(3) Specificity can evaluate the correct identification ability of the model for abnormal samples. It is calculated as follows:
  (4) $$Specificity=\frac{\mathrm{TN}\;}{TN+FP}\times 100\%$$
(4) Precision refers to the ratio of the number of correctly classified normal samples to the number of samples that are classified as normal samples and can be calculated as follows:
  (5) $$Precision=\frac{\mathrm{TP}\;}{TP+FP}\times 100\%$$

(TP): True positive; (FP): False Positive; (TN): True negative; (FN): False negative.

## 3. Results and Discussion

### 3.1. The Original and SNV Pre-Processed Spectra

**Figure 3:**
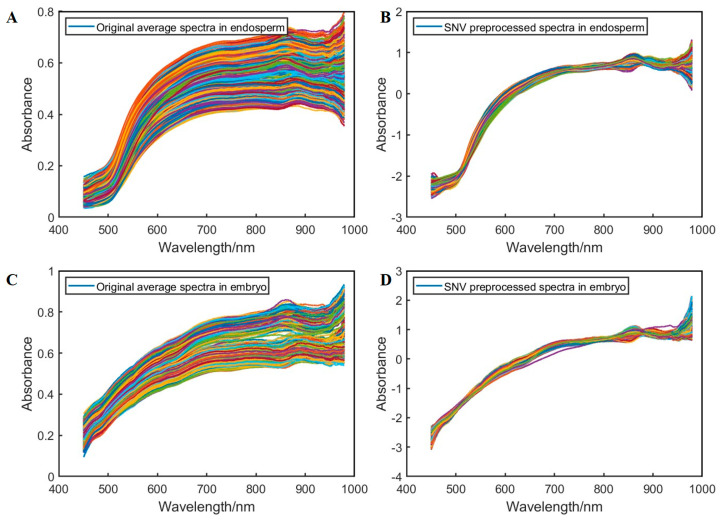
The average spectra of different freeze-damaged corn seed samples. (**A**) Original average spectra on the endosperm side; (**B**) SNV preprocessed spectra on the endosperm side; (**C**) Original average spectra on the embryo side; (**D**) SNV preprocessed spectra on the embryo side.

Figure 3 shows the original average spectra and SNV pre-processed spectra of three-category freeze-damaged corn seed samples in endosperm (A, B) and embryo (C, D).

### 3.2. The Results in the Endosperm and Embryo Side

#### 3.2.1. Confusion and Accuracy

Table 1 shows the confusion matrix and accuracy results of the SVM and DCNN models on the endosperm side with non-pretreatment and SNV pretreatment methods. From Table 1, it could be seen that the accuracy of both models was generally better on the training set than on the testing set, which indicated that the constructed model did not have a significant overfitting phenomenon to a certain extent. Specifically, the DCNN model showed a higher classification accuracy than the SVM model. The accuracy of the training set and testing set of the DCNN model was 96.6% and 92.8%, respectively, which was 3.0% and 4.2% higher than that of the SVM model (93.6% and 88.6%) under no pretreatment. Under SNV pretreatment, the accuracy of the training set and testing set of the DCNN model reached 96.9% and 93.8%, respectively, which was 4.0% and 3.8% higher than that of the SVM model of 92.9% and 90.0%, respectively. It could be seen that the SNV pretreatment method can obtain better classification results than no pretreatment. Further analysis of the confusion matrix data showed that only a small amount of misclassification occurred in the corn seed samples of category 1, while there was a significant misclassification between the seed samples of category 2 and category 3, which may be related to the freezing damage suffered by both types of seeds.

Table 2 shows the confusion matrix and accuracy results of the SVM and DCNN models under the non-pretreatment and SNV pretreatment methods on the embryo side. It can be seen from the confusion matrix that both models performed well in the classification of corn seed samples of freezing damage category 1, and all correct classifications were realized. However, there were some misclassifications in the classification of seed samples of category 2 and category 3. In terms of accuracy, the DCNN model performed better than the SVM model. Specifically, the accuracy of the DCNN model on the testing set without pretreatment was 96.9%, which was 2.8% higher than that of the SVM model (94.1%). At the same time, the testing set accuracy of the DCNN model was 96.7%, which was 1.3% higher than that of the SVM model (95.4%) under SNV pretreatment. Comparing the results before and after pretreatment, it was found that the accuracy of the SVM model was improved after SNV pretreatment, the accuracy of the training set was increased from 97.3% to 98.1%, and the accuracy of the test set was increased from 94.1% to 95.4%. In contrast, the accuracy of the DCNN model in both the training set and the testing set did not change much, and it showed good performance. Compared with the results of the endosperm surface of corn seeds in Table 1, the results of the embryo surface showed a significant improvement, and the number of misclassifications was significantly reduced. Furthermore, from the perspective of accuracy value, the highest accuracy of the DCNN model in the endosperm surface testing set was 93.8%, while the accuracy of the embryo surface model was 96.7%, indicating that hyperspectral data based on the embryo surface had higher accuracy in discriminating seed freezing damage, which may be related to the characteristics of seed embryos that can reflect the related conditions of seeds.

#### 3.2.2. Sensitivity

**Figure 4:**
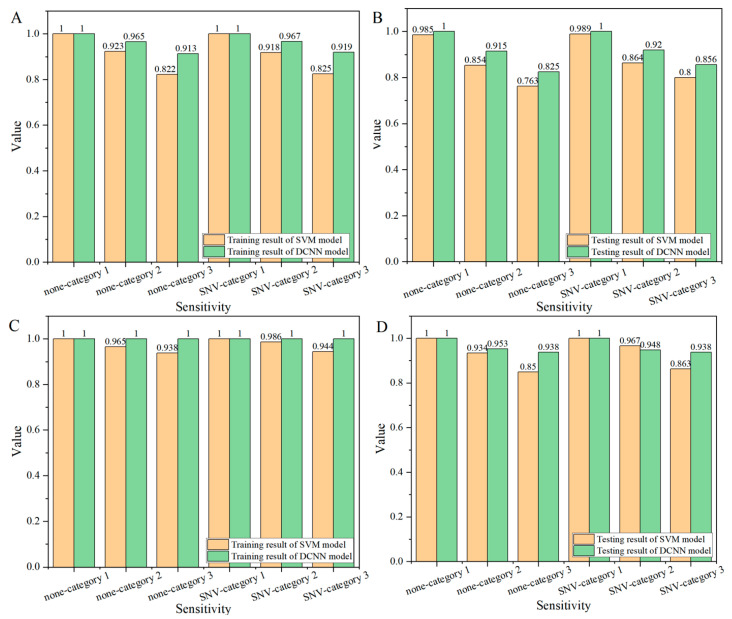
The classification sensitivity results (orange for the SVM model, green for the DCNN model) of corn seeds: (**A**) training set on the endosperm side, (**B**) testing set on the endosperm side, (**C**) training set on the embryo side, (**D**) and testing set on the embryo side.

Sensitivity is defined as the probability that a sample of a category is correctly classified. Figure 4 shows the classification sensitivity results of corn seeds on the endosperm side (Figure 4A,B) and the embryo side (Figure 4C,D). According to Figure 4A,B, the classification sensitivity of samples of freezing damage category 1 was higher than that of the other two categories, while the classification sensitivity of samples of category 3 was the lowest. In the SVM model, the classification sensitivity of the training set of frost damage category 1 reached 100%, the lowest classification sensitivity of the testing set was 98.5% (Figure 4B, no pretreatment), the classification sensitivity of category 2 was relatively low, and the lowest classification sensitivity of the training set was 91.8% (Figure 4A, SNV pretreatment). However, the highest classification sensitivity of the testing set was only 86.4% (Figure 4B, SNV pretreatment), and the classification sensitivity of category 3 was the lowest, and the classification sensitivity of the testing set was less than 80%. As shown in Figure 4A,B, the classification results of the DCNN model were significantly better than those of the SVM model, and the classification sensitivity values of both category 2 and category 3 were improved. The classification sensitivity value of the testing set of category 2 was higher than 91.5% (Figure 4B, no pretreatment). The testing set of category 3 had a minimum classification sensitivity value of 82.5% (Figure 4B, no pretreatment).

In the analysis of the embryo (Figure 4C,D), the modeling performance of the DCNN model was significantly better than that of the SVM model. Similar to the results of the endosperm side, the sensitivity of category-1 samples was higher than the other two categories, especially the sensitivity value of category 3 samples was the lowest. However, it can be seen from the figure that the sensitivity of category 3 samples had been significantly improved compared with the endosperm surface. Taking the testing set as an example, the sensitivity of category 3 samples in the SVM model can reach up to 86.3% (Figure 4D, SNV pretreatment), while in the DCNN model, the sensitivity value of category 3 samples could reach 93.8%, and the sensitivity value was at least 7% higher. Compared with the endosperm side, the sensitivity of the corn seed on the embryo side was significantly improved, especially for category 2 (the sensitivity of the SVM and DCNN models of the testing set improved by at least 8% (no pretreatment) and 2.8% (SNV pretreatment)) and category 3 (the sensitivity of the SVM and DCNN models of the testing set improved by at least 6.3% (SNV pretreatment) and 8.2% (SNV pretreatment)).

#### 3.2.3. Specificity

**Figure 5:**
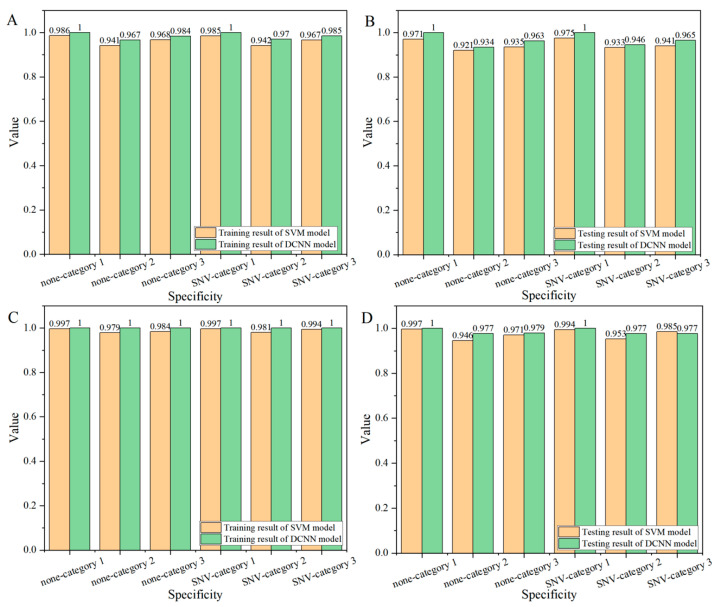
The classification specificity results (orange for the SVM model, green for the DCNN model) of corn seeds: (**A**) training set on the endosperm side, (**B**) testing set on the endosperm side, (**C**) training set on the embryo side, and (**D**) testing set on the embryo side.

Specificity can reveal the degree of differentiation between different categories, and the greater the difference between categories, the higher the specificity value. Figure 4 showed the specificity results of corn seeds on the endosperm side (Figure 5A,B) and embryo side (Figure 5C,D). According to Figure 5A,B, the specificity of category 1 was higher than that of other categories on the endosperm side, followed by category 3, and category 2 had the lowest specificity. Based on this index, the specificity values of SVM and DCNN models both exceeded 90%, in which the SVM model had the lowest specificity of 92.1% in the testing set (Figure 5B, SNV-Category 2), while the DCNN model had the lowest specificity of 93.4% (Figure 5B, SNV-Category 2). In all cases, the specificity values of the DCNN model were higher than those of the SVM model, which indicated that the modeling performance of the DCNN model was better than that of the SVM model.

In the analysis of the embryo (Figure 5C,D), the specificity values of category 1 were higher than those of the other two categories. The specificity values of SVM and DCNN models were both higher than 94%. The specificity value of the DCNN model was higher than that of the corresponding SVM model under different cases. The specificity of the training set of the DCNN model reached 100%, while the specificity of the testing set was the lowest at 97.7%, indicating that the modeling result of the DCNN model was better than that of the SVM model. Compared with the specificity of the endosperm side, the result of the embryo was greatly improved, indicating that the embryo surface could better explain the difference between frozen damaged seeds.

#### 3.2.4. Precision

**Figure 6:**
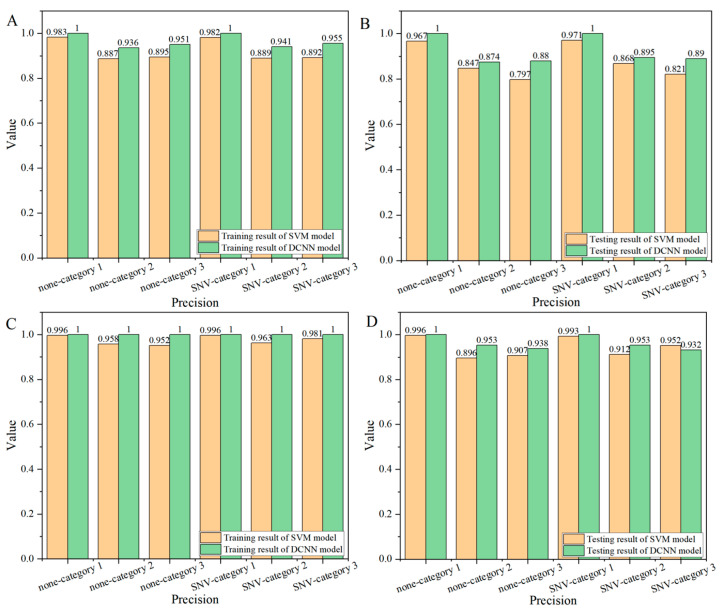
The classification precision results (orange for the SVM model, green for the DCNN model) of corn seeds: (**A**) training set on the endosperm side, (**B**) testing set on the endosperm side, (**C**) training set on the embryo side, and (**D**) testing set on the embryo side.

Figure 5 shows the precision analysis results of the endosperm side (Figure 6A,B) and embryo side (Figure 6C,D) of corn seeds. According to Figure 6A,B, the classification precision of category 1 samples on the endosperm side was significantly higher than that of samples of category 2 and category 3. The precision value of the category-1 testing set of the SVM and DCNN models was higher than 96.7% (SVM model—no pretreatment). In the SVM model, the highest precision value of category-2 testing set was up to 86.8% (Figure 6B, SNV pretreatment), while the highest precision value of the category-3 samples was only 82.1% (Figure 6B, SNV pretreatment). Compared with the SVM model, the precision values of category 2 and category 3 of the DCNN model had been improved. The precision value of category-2 samples exceeded 87.0%, and the lowest was 87.4% (Figure 6B, no pretreatment). The precision value of category 3 samples was higher than 88.6% (Figure 6B, no pretreatment). Comprehensive analysis showed that the modeling performance of the DCNN model was better than that of the SVM model.

In the analysis of the embryo (Figure 6C,D), the precision value of category 1 samples was higher than that of category 2 and category 3 samples. Compared with the SVM model, the precision value of both category 2 and category 3 by the DCNN model had improved. In the SVM model, the precision value of the category-2 testing set reached the highest 91.2% (Figure 6D, SNV pretreatment), while the accuracy of the category-2 testing set in the DCNN model was 95.3%, 4.1% higher than the former. Obviously, the modeling result of the DCNN model was better than that of the SVM model. Compared with the precision results of the endosperm side, the results of the embryo side of corn seeds were significantly improved, especially for corn seeds of category 2 and category 3. Compared with the precision value of the testing set on the endosperm side for category 2, the SVM model improved at least 4.4% (SNV pretreatment), and the DCNN model improved at least 4.8% (SNV pretreatment). At the same time, compared with the precision value of the testing set on the endosperm side for category 3, the SVM model improved by at least 11% (no pretreatment), and the DCNN model improved by at least 4.4% (SNV pretreatment).

### 3.3. Discussion

From the above modeling results, the DCNN model achieved better performance in accuracy, sensitivity, specificity, and accuracy indicators than the SVM model. The reason can be explored by the principles of these two models, which have the difference of feature learning ability. Compared with the DCNN model, SVM relies on manual feature extraction and cannot automatically learn deep features. DCNN can automatically learn multiple layers of features through a multi-layer structure, from the bottom layer to the top layer, and improve the classification accuracy. At the same time, DCNN performs well on large-scale data, and the increase in data volume helps to learn rich features, alleviate overfitting, and improve generalization ability and accuracy.

From the classification results of the endosperm and embryo sides of corn seeds, the classification results of the embryo side were better than those of the endosperm side, indicating that the embryo-side hyperspectral image data could better reflect the freezing damage of seeds to a certain extent. It can be known that seed embryos play a vital role in the growth and development of seeds. It contains a higher concentration of metabolically active substances, organelles, and membrane systems, which are more vulnerable to damage during freezing, which may lead to the breakdown and loss of function of the membrane and the texture of the seed coat [3].

## 4. Conclusions

In this study, DCNN was introduced to identify freezing damage on the endosperm side and embryo side of single corn seeds based on hyperspectral imaging technology. Multiple evaluation indexes (including accuracy, sensitivity, specificity, and precision) were used to comprehensively evaluate the performance of the SVM and DCNN models in the whole waveband, and the classification results of corn seeds were compared and analyzed.

Compared with the SVM model, the DCNN model showed better performance in accuracy, sensitivity, specificity, and accuracy. In the absence of pretreatment, the accuracy value of the testing set in the endosperm and embryo DCNN model was increased by 4.2% and 2.8%, respectively. Under the condition of SNV pretreatment, the accuracy value of the testing set in the endosperm and embryo DCNN model was improved by 3.8% and 1.3%, respectively. In addition, the classification results of the embryo-side corn seeds were better than those of the endosperm side, in which the seeds of freezing damage category 1 on the embryo side could be fully recognized. The accuracy value of the testing set of the DCNN model on the embryo side was higher than 96.7%, while the accuracy value of the DCNN model on the endosperm side was lower than 93.8%. Compared with the sensitivity results, the sensitivity value of the category-2 testing set of the embryo-side SVM model increased by at least 8%, and that of the DCNN model increased by at least 2.8%. The sensitivity value of the category-3 testing set of the SVM model was improved by at least 6.3%, and the DCNN model was improved by at least 8.2%. Compared with the specificity and precision of endosperm side, the results of the embryo side of the corn seed showed significant improvement, which indicated that embryo-side data could reflect the freezing damage of seeds more accurately to a certain extent. This study proved that the DCNN model can accurately and quickly classify endosperm and embryo corn seeds of different categories of freezing damage, which provided a theoretical basis for constructing an end-to-end recognition and classification model of frozen corn seeds.

Although this study fills the gap of different side detection of corn seed freezing damage and obtains the relevant conclusions. However, there are also some limitations to research. For example, although high accuracy results can be obtained, hyperspectral imaging equipment is often expensive and large in size, which limits its wide application in large-scale field freezing damage detection, and the prepared samples only reflect relevant situations as much as possible and cannot fully reflect the complex actual situation in the field. In the aspect of data analysis, the amount of hyperspectral data is huge, and sometimes it is difficult to realize real-time freezing damage detection. Although the image processing method is used to separate ROIs, the spatial information is not well utilized. If the spatial and spectral information is fused, the performance of the model may be improved to some extent. In future research and development, there will be a need to explore how the findings can be applied in practice. For example, in real life, in order to ensure the detection speed when suffering from freezing damage, it is necessary to establish a general identification model of the seed embryo and the endosperm side. Meanwhile, in the follow-up research, the feature wavelength of the spectrum can be extracted to establish a fast and non-destructive multi-spectral detection system for frozen corn seeds.

## Figures and Tables

**Table 1:** The confusion matrix and accuracy results of the SVM and DCNN models under the non-pretreatment and SNV pretreatment methods on the endosperm side.

Pretreatment	Samples	Category	SVM			DCNN		
			Category 1	Category 2	Category 3	Category 1	Category 2	Category 3
none	Training set	Category 1	533	/	/	533	/	/
		Category 2	2	394	31	/	412	15
		Category 3	7	50	263	/	28	292
		Accuracy	93.0%			96.6%		
	Testing set	Category 1	263	1	3	267	/	/
		Category 2	3	182	28	/	195	18
		Category 3	6	32	122	/	28	132
		Accuracy	88.6%			92.8%		
SNV	Training set	Category 1	533	/	/	533	/	/
		Category 2	3	392	32	/	413	14
		Category 3	7	49	264	/	26	294
		Accuracy	92.9%			96.9%		
	Testing set	Category 1	264	1	2	267	/	/
		Category 2	3	184	26	/	196	17
		Category 3	5	27	128	/	23	137
		Accuracy	90.0%			93.8%		

**Table 2:** The confusion matrix and accuracy results of SVM and DCNN models under the non-pretreatment and SNV pretreatment methods on the embryo side.

Pretreatment	Samples	Category	SVM			DCNN		
			Category 1	Category 2	Category 3	Category 1	Category 2	Category 3
none	Training set	Category 1	533	/	/	533	/	/
		Category 2	/	412	15	/	427	/
		Category 3	2	18	300	/	/	320
		Accuracy	97.3%			100%		
	Testing set	Category 1	267	/	/	267	/	/
		Category 2	/	199	14	/	203	10
		Category 3	1	23	136	/	10	150
		Accuracy	94.1%			96.9%		
SNV	Training set	Category 1	533	/	/	533	/	/
		Category 2	/	421	6	/	427	/
		Category 3	2	16	302	/	/	320
		Accuracy	98.1%			100%		
	Testing set	Category 1	267	/	/	267	/	/
		Category 2	/	206	7	/	202	11
		Category 3	2	20	138	/	10	150
		Accuracy	95.4%			96.7%		

## Data Availability

The original contributions presented in this study are included in the article. Further inquiries can be directed to the corresponding author.
